# Body Mass Index and Its Association with Genetically Transmitted Traits

**DOI:** 10.1155/2020/3469316

**Published:** 2020-12-21

**Authors:** Sultan Z. Alasmari, Nashwa Eisa, Saeed Mastour Alshahrani, Mohammad Mahtab Alam, Prasanna Rajagopalan, Mohammed Makkawi

**Affiliations:** ^1^Department of Clinical Laboratory Sciences, Faculty of Applied Medical Sciences, King Khalid University, Guraiger, Abha 62529, Saudi Arabia; ^2^Department of Basic Medical Sciences, College of Applied Medical Sciences, King Khalid University, Guraiger, Abha 62529, Saudi Arabia

## Abstract

**Background:**

Body mass index (BMI) is a metric widely used to measure the healthy weight of an individual and to predict a person's risk of developing serious illnesses. Study the statistical association between genetically transmitted traits and BMI might be of interest.

**Objectives:**

The present study designed to extend the inadequate evidence concerning the influence of some genetically transmitted traits including ABO blood type, Rh factor, eye color, and hair color on BMI variation.

**Methods:**

A total of 142 undergraduate female students of the Department of Clinical Laboratory Sciences, Faculty of Applied Medical Sciences, King Khalid University, Abha, Saudi Arabia, were participated to investigate the possible linkage between genetic traits and BMI variations. Height and weight are collected from participants for BMI measurement. ABO blood type and Rh factor were determined by antisera.

**Results:**

Out of 142 female students, 48 were categorized in the first tertile (T1: less than 19.8 kg/m^2^), 50 were categorized in the second tertile (T2: between 19.8 and 23.7 kg/m^2^), and 44 were categorized in the third tertile (T3: greater than 23.7 kg/m^2^). Chi-square analysis shows that there were no associations of genetic traits including hair color, eye color, ABO blood type, and Rh blood type with BMI. However, a significant association between hair color and BMI was observed using multinomial logistic regression analysis.

**Conclusions:**

Our data provides a more robust prediction of the relative influence of genetic effects such as hair color on BMI. Future studies may contribute to identifying more association between genes involved in hair pigmentation and BMI variation.

## 1. Introduction

The aetiology of the diseases are varied, but the involvement of family history may consider one of the common risk factors of numerous diseases, including cancer, cardiovascular disease (CVD), diabetes, autoimmune disorders, and psychiatric illnesses [1]. Personal attitude and human genes transferred behaviorally and genetically by parents and may contribute to well-being and personal satisfaction [1, 2]. Human well-being is achieved by factors involving economy, health, live events, and social relationship [2]. Besides, a genetically transmitted trait such as hair color and eye color involves in human attractiveness and promotes personal satisfaction. Yet, the knowledge of the association between personality and life satisfaction with genetic and environmental factors remains limited [2].

Obesity and overweight are the most common lifestyle diseases, affecting approximately 40% of adults globally [3]. Body mass index (BMI) is a common clinical measurement for these issues that can be easily calculated with no equipment, once an individual's weight and height are known. According to the World Health Organization (WHO), the results of BMI calculations can be grouped into several categories: underweight (BMI < 18.5 kg/m^2^), normal (18.5 ≤ BMI < 25 kg/m^2^), overweight (25 ≤ BMI < 30 kg/m^2^), and obese (BMI ≥ 30 kg/m^2^) [4].

Although environmental and behavioral factors such as high-calorie food consumption and low physical activity may contribute to obesity, genetic factors are also involved in gaining weight [3, 5]. Family and twin studies have determined that individuals with genetic differences show a variation in body mass index (BMI) [6–8]. Being overweight or obese is a significant risk factor for developing some chronic diseases and disorders which contribute to increased mortality, but weight loss can reduce the risk [9], although increasing energy expenditure by physical activity and reducing calorie intake by diet have a positive impact on weight loss and lead to positive health consequences [10]. A complex interaction of factors such as genetic, developmental, behavioral, and environmental has shown to influence body weight and fat mass variation [11]. For instance, urbanization has revealed to be a risk factor for developing obesity worldwide, which indicates the involvement of the environment. Additionally, twin studies have shown that genetics and obesity linkage are related behaviors including eating patterns and exercise [11].

In contrast, little is known about the association between specific genetic characteristics and thin individuals [5]. A study on 7078 UK children and adolescents demonstrated a correlation between child/adolescent thinness and parental weight status. The prevalence of thinness was higher when the parents were thin than when they were normal weight, overweight, or obese [5, 12]. To develop a future antiobesity target drug, a detailed understanding of the mechanisms underlying thinness/resistance to obesity is needed [5].

The present study is aimed at determining the possible linkage concerning the influence of some genetically transmitted traits including ABO blood type, Rh factor, eye color, and hair color on BMI variation.

## 2. Methodology

### 2.1. Participants

This study was carried out among 142 undergraduate female students of the Clinical Laboratory Sciences, Faculty of Applied Medical Sciences, King Khalid University, Abha, Saudi Arabia. The age of the participated students ranged between 19 and 29 years old, with a mean of 20.77.

### 2.2. Blood Type and the Rh Factor Analysis

Blood samples were obtained by pricking the finger with a lancet. The drop of blood was placed on a transparent glass slide and mixed with the antisera (anti-A, anti-B and anti-D) using wooden sticks to determine agglutination.

### 2.3. Measures

The participants were asked to complete a questionnaire that required the filling in of details regarding their height, weight, natural eye color, and natural hair color. Their BMI were then measured, and their natural eye and hair color recorded, with the help of the completed questionnaires. Based on their BMI, the participants were then categorized in tertiles to ensure approximate allocations of subjects per group as follows: T1: ≤19.8 kg/m^2^, T2: 19.8–23.7 kg/m^2^, and T3>23.7 kg/m^2^.

### 2.4. Ethical Consideration

Ethical approval (approval number: (ECM#2020-197)—(HAPO-06-B-001)) for the study was obtained from the Ethical Committee of Scientific Research, King Khalid University. The participants were reassured that the information will be used only for research purposes.

### 2.5. Statistical Analysis

Sample characteristics are reported as mean with standard deviation (SD) for continuous variables and number of subjects (*n*) as a percentage (%) (Table 1). We used the Chi-square, particularly Fisher's exact test, to assess the relationship between BMI and the inheritance of several genetic factors. Correlation analysis was also used by calculating Spearman correlation coefficient. We also used multinomial logistic regression to model the association between BMI and genetic inheritance. Model assumptions including linearity, independent observation, and absence of multicollinearity were checked. No violations of assumptions were observed.

The levels of the genetic inheritance of several traits such as hair color, eye color, and ABO blood type were collapsed into two levels to maintain sufficient power in the analysis. The covariates selected were hair color (black vs. nonblack), eye color (brown vs. nonbrown), ABO blood type (O vs. others), and Rh blood type (positive vs. negative). We also controlled for age as it may be a predictor of BMI. SPSS version 21.0 software (SPSS Inc., Chicago, IL, USA) was used to perform the current analyses.

### 2.6. Results

Out of 142 female students, 48 were categorized in the first tertile (T1: less than 19.8 kg/m^2^), 50 were categorized in the second tertile (T2: between 19.8 and 23.7 kg/m^2^), and 44 were categorized in the third tertile (T3: greater than 23.7 kg/m^2^). First tertile group was seen to have more black-hair colored subjects, as compared with second and third tertiles. In addition, the first tertile group saw more subjects with brown eye color and a high prevalence of “O” blood type, as compared with second and third tertiles. Age and Rh blood type appeared to be approximately similar across all the groups (Table 1).

Results from Chi-square show that there were no associations of genetic traits including hair color, eye color, ABO blood type, and Rh blood type with BMI (*χ*^2^ = 4.626; df = 2; *P* value = .102 for hair color; *χ*^2^ = 1.964; df = 2; *P* value = .373 for eye color; *χ*^2^ = 0.320; df = 2; *P* value = .864 for ABO blood type; *χ*^2^ = 1.013; df = 2; *P* value = .702 for Rh blood type). Furthermore, Spearman correlation coefficients did not provide evidence that genetic traits including hair color, eye color, ABO blood type, and Rh blood type were associated with BMI (*r* = 0.145; *P* value = .085 for hair color; *r* = 0.066; *P* value = .432 for eye color; *r* = 0.045; *P* value = .591 for ABO blood type; *r* = –0.073; *P* value = .392 for Rh blood type) (Table 2).

To further investigate the association of BMI with these genetic inheritances, results from multinomial logistic regression were reported as odds ratio (OR) and at 95% CI in Table 3. There were no associations of eye color, ABO blood type, and Rh blood type with BMI. However, there was an association between hair color and BMI. That is, compared with nonblack hair color females, those with black hair color were less likely to have weight less than 19.8 kg/m^2^, which is the first tertile weight (OR: 0.41; 95% CI: 0.18–0.94; *P* value = .035). Specifically, females with black hair color were more likely to be in the second or the third tertile of BMI levels (Table 3).

## 3. Discussion

Genetic influences on traits can be studied by various methods [13]. However, twin and adoption studies are most commonly used. In the present study, a simpler alternative method was used for assessing the correlation of BMI with blood groups, eye, and hair pigmentation. There is a distinct phenotype-genotype correspondence among eye color, natural hair color, blood type, and the Rh factor. To this end, we conducted an original study by collecting blood samples to determine the blood group types and providing questionnaires to participants for obtaining information regarding eye color and hair color, also on height and weight information for BMI measurements. This study is aimed at assessing the correlation between some genetically transmitted traits, including blood groups, eye, and hair pigmentation with BMI variations among an adult female population. Results from Chi-square, Spearman correlation coefficients, and multinomial logistic regression revealed no statistical differences between eye color, ABO blood type, and Rh blood type with the variation of BMI. Although Chi-square and Spearman correlation coefficients did not show a significant association between hair color and BMI, multinomial logistic regression analysis showed a statistical correlation between hair color and BMI.

To the best of our knowledge, no studies investigated the linkage between BMI and hair color. However, several studies carried out in determining the correlation between hair color and disease states. A study carried on Czech and Slovak population revealed that health status in red-haired women was the most divergent comparison to other color haired women [14]. Additionally, a large-volume study conducted on US population showed that risk factor for developing Parkinson's disease increases with decreasing darkness of hair color [15]. Moreover, a recent report revealed that there is no direct effect of hair cortisol and hair cortisone on BMI [16].

It is evident that obesity results from an interaction between genetic and environmental factors. Tanning ability, hair color, skin color, and eye color are typical visible human pigmentation traits of which their regulation involves a handful of pigmentation genes [17]. To this context, a tight correlation between melanosomes and hair color was recently reported [18]. Melanocyte-stimulating hormones (MSHs) and derived proopiomelanocortin (POMC) protein were reported to have a greater influence on the biological effects by activating the melanocortin receptors (MCRs) belonging to the family of G-protein-coupled seven-transmembrane receptors [19]. Additionally, *α*-MSH signals via MC1R were shown responsibility in determining skin and hair pigmentation [20, 21]. Further, MC4R is constitutively expressed in the hypothalamus and is involved in the regulation of feeding behavior, energy homeostasis, and obesity development [22, 23]. At the presents, at least five MCRs have been cloned and identified, among which there is a high sequence homology with a shared signaling pathway [24].

Discovering agouti gene dominant mutations which are shown to lead to yellow fur, adult-onset obesity, insulin resistance, and increased susceptibility to cancer in mice could assist to trace back the evidence of the genetic association between obesity and pigmentation [25]. In addition, the relationship between obesity and genetic defects has been determined. Krude et al. study revealed a correlation between red hair color with POMC mutations and early-onset obesity [26]. These mutations were either interferes with the synthesis of *α*-MSH or abolish POMC translation [26]. Studies also show that PPAR-*γ* coactivators, the key regulators of energy metabolism, regulate the transcription of MITF and melanin production in response to *α*-MSH in melanocytes [27]. The same study demonstrated that the genetic variants in PPAR-*γ* coactivators are associated with human tanning, which provides a novel link between energy metabolism and pigment formation [27].

Having said that, detailed studies are needed to verify the association of BMI with genetically transmitted traits of pigmentation and their relation to phenotypic alterations such as the hair color.

## 4. Conclusion

In conclusion, the undeniable reports that demonstrated the association between genetic and disease permits for expanding the exploration toward the possible linkage connecting genetic traits and BMI. Despite the data showed no significant correlation between BMI variation and ABO blood group, Rh system, eye color and hair color in some analysis, multinomial logistic regression analysis showed an association between BMI and hair color. This finding may shed light on the importance of further investigating the mechanisms behind this association. The present study focused on the correlation between BMI variations and some genetically transmitted traits of a small adult female population. This factor limited the scope of our research, evidencing the need for future studies with a larger number of subjects involving male participants as well.

## Figures and Tables

**Table 1:** Sample characteristics (*N* = 142).

Variable	BMI (kg/m^2^)		
	T1: <19.8 (*n* = 48)	T2: 19.8–23.7 (*n* = 50)	T3: >23.7 (*n* = 44)
Age, mean (SD)	20.5 (0.8)	20.7 (1.2)	20.9 (1.7)
Body mass index, mean (SD)	18.3 (1.1)	21.9 (1.1)	27.1 (3.2)
Hair color, *n* (%)			
Black	28 (58.3)	19 (38.0)	18 (40.9)
Brown	7 (14.6)	6 (12.0)	7 (15.9)
Dark brown	13 (27.1)	25 (50.0)	19 (43.2)
Eye color, *n* (%)			
Blue/grey/green	0 (0.0)	1 (2.0)	0 (0.0)
Brown	44 (91.7)	41 (82.0)	38 (86.4)
Hazel	4 (8.3)	8 (16.0)	6 (13.6)
ABO blood type, *n* (%)			
A	13 (27.1)	17 (34.0)	16 (36.4)
AB	1 (2.1)	0 (0.0)	1 (2.3)
B	3 (6.3)	2 (4.0)	1 (2.3)
O	31 (64.6)	31 (62.0)	26 (59.1)
Rh blood type, *n* (%)			
Rh-positive	45 (93.8)	47 (94.0)	43 (97.7)
Rh-negative	3 (6.3)	3 (6.0)	1 (2.3)

**Table 2:** Association of BMI with genetic traits^1^.

Variable	BMI (kg/m^2^)			*χ* ^2^ value	DF	*P* value^2^	Correlation coeffecient^3^	*P* value^2^
	T1: <19.8	T2: 19.8–23.7	T3: >23.7					
Hair color, *n* (%)				4.626	2	.102	0.145	.085
Black	28 (58.3)	19 (38.0)	18 (40.9)					
Nonblack	20 (41.7)	31 (62.0)	26 (59.1)					
Eye color, *n* (%)				1.964	2	.373	0.066	.432
Brown	44 (91.7)	41 (82.3)	38 (86.4)					
Nonbrown	4 (8.3)	9 (18.0)	6 (13.6)					
ABO blood type, *n* (%)				0.320	2	.864	0.045	.591
O	31 (64.6)	31 (62.0)	26 (59.1)					
Others	17 (35.4)	19 (38.0)	18 (40.9)					
Rh blood type, *n* (%)				1.013	2	.702	–0.073	.392
Positive	45 (93.8)	47 (94.0)	43 (97.7)					
Negative	3 (6.3)	3 (6.0)	1 (2.3)					

^1^Results were reported from Chi-square (Fisher's exact test) and Correlation analyses. ^2^Level of significance is at *α* = 0.05. ^3^Spearman Correlation.

**Table 3:** Multinomial logistic regression of the association of BMI with genetic traits^1,2^.

Variable	BMI (kg/m^2^)		
	T1^3^: <19.8	T2: 19.8–23.7	T3: >23.7
Hair color	1.00	0.41 (0.18–0.94)	0.50 (.21–1.17)
	*P* value	.035	.109
Eye color	1.00	0.34 (0.09–1.24)	0.48 (.12–1.91)
	*P* value	.102	.300
ABO blood type	1.00	1.00 (0.43–2.36)	0.92 (.38–2.21)
	*P* value	.997	.851
Rh blood type	1.00	0.93 (0.17–5.01)	2.51 (.25–25.40)
	*P* value	.929	.436
Age	1.00	1.15 (0.81–1.65)	1.21 (.85–1.74)
	*P* value	.437	.286

^1^Results are reported as odds ratio (OR) and 95% confidence interval (CI) and *P* value. ^2^Independent variables included in the model are hair color (black vs. nonblack), eye color (brown vs. nonbrown), ABO blood type (O vs. others), Rh blood type (positive vs. negative) and age (continuous). ^3^Used as reference group.

## Data Availability

Raw data were generated at the Department of Clinical Laboratory Sciences, Faculty of Applied Medical Sciences, King Khalid University. Derived data supporting the findings of this study are available from the corresponding author Sultan Z. Alasmari on request.
